# Detection of hemorrhage by analyzing shapes of the arterial blood pressure waveforms

**DOI:** 10.1186/2197-425X-3-S1-A589

**Published:** 2015-10-01

**Authors:** S Romero Zambrano, M Guillame-Bert, A Dubrawski, G Clermont, MR Pinsky

**Affiliations:** Carnegie Mellon University, H. John Heinz III College, Pittsburgh, PA USA; Carnegie Mellon University, School of Computer Science, Pittsburgh, PA USA; University of Pittsburgh, School of Medicine, Pittsburgh, PA USA

## Introduction

We hypothesize that changes of shape of arterial blood pressure (ABP) high-frequency waveform signal can be reflective of body's response to stress, in particular to hemorrhage.

## Objective

To apply Machine Learning (ML) and sequential pattern extraction to ABP waveforms to reliably detect slow bleeding.

## Methods

62 healthy pigs are anesthetized, intubated, ventilated, and instrumented prior to and during a controlled bleeding at a rate of 20 ml/min. ABP is recorded at a 250 Hz rate. To estimate the likelihood of bleeding, our algorithm:

(1) Extracts 10 s disjoint intervals of ABP waveform and standardizes the time series to zero mean and unit standard deviation;

(2) Discretizes the standardized data into a sequence of symbols each reflective of a particular value of the standardized ABP;

(3) Identifies which sequential patterns of symbols are present in the current interval data from among patterns previously extracted from training data using SPADE algorithm [1];

(4) Feed the identified sequential patterns into previously trained ML classifier (Random Forest) to predict current bleeding status of the subject. The procedure is empirically evaluated using leave-one-pig-out crossvalidation.

## Results

**Figure 1:**
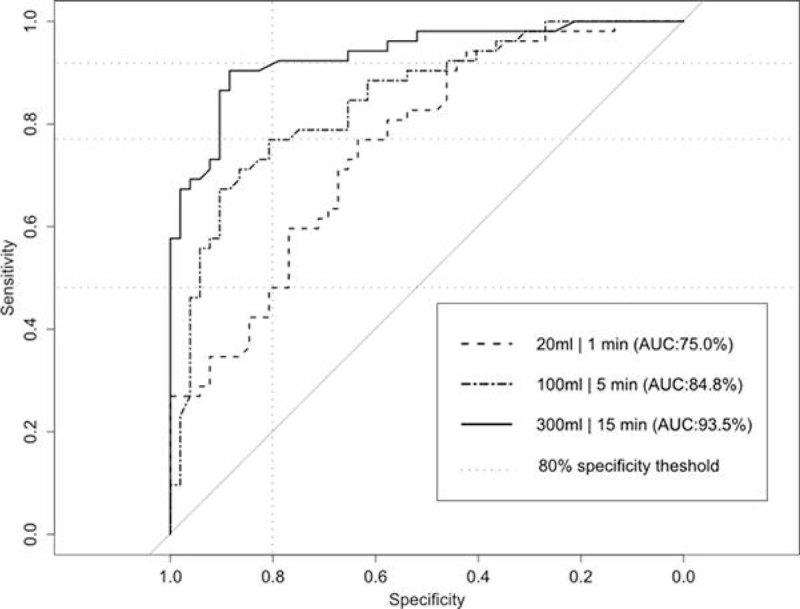
**ROC of bleeding detection model at 1, 5, and 15 min from the onset of controlled hemorrhage.**

Figure 1 shows crossvalidation ROC curves for 1 min, 5 min and 15 min time marks after the onset of bleeding. At 80% specificity, our approach detects 42% of bleeding pigs at just one minute into bleeding (20 ml of blood lost, 75% AUC), 73% of bleeding pigs at 5 min (100 ml of blood lost, 85% AUC), and 90% of bleeding pigs at 15 min (300 ml of blood lost, 94% AUC).

**Figure 2:**
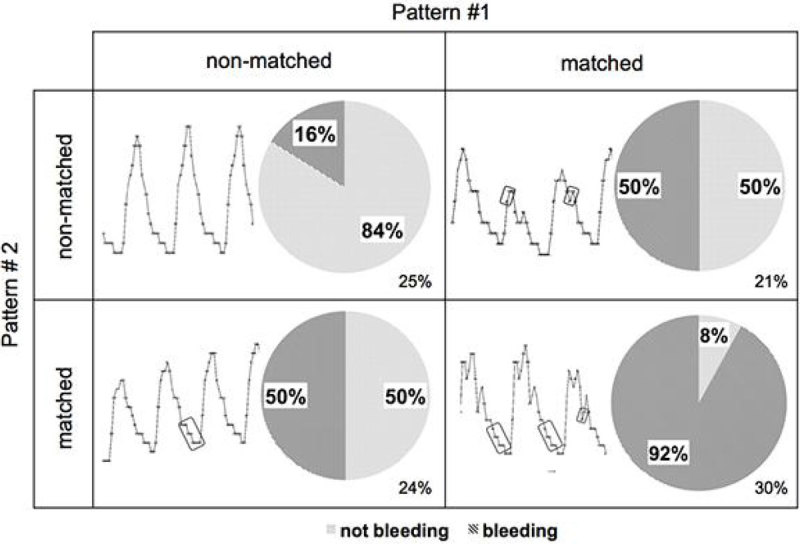
**Distribution of probability of bleeding based on the two most informative sequential patterns of shape of the ABP waveform.**

In addition, our method enables easy interpretation of learned patterns. An example in Figure 2 shows how distribution of bleed vs. no-bleed test cases changes when two most informative sequential patterns are present or absent in ABP waveform. When both patterns are absent, the probability that the subject is bleeding is 16%. When either is present the probability increases to 50%, and if both are present it reaches 92%. Either of these patterns is present in 54% of all data. Figure 2 also shows representative ABP waveforms for the four combinations of the absence and presence of these patterns.

## Conclusions

ML and sequential pattern extraction enables effective monitoring of ABP waveforms for indications of hemorrhage. The presented approach can be used to enhance current practice of hemodynamic monitoring while helping clinicians interpret patterns of patients' physiological response to stress.

## Acknowledgments

Work partially supported by NSF (award 1320347) and by NIH (grant NR013912).

## References

[1] Zaki MJ (2001). SPADE: An efficient algorithm for mining frequent sequences. Machine Learning.

